# Premature Ovarian Insufficiency - an update on recent advances in understanding and management

**DOI:** 10.12688/f1000research.11948.1

**Published:** 2017-11-29

**Authors:** Saioa Torrealday, Pinar Kodaman, Lubna Pal

**Affiliations:** 1 Division of Reproductive Endocrinology and Infertility, Department of Obstetrics & Gynecology, Womack Army Medical Center, Fort Bragg, North Carolina, USA; 2 Division of Reproductive Endocrinology and Infertility, Department of Obstetrics, Gynecology and Reproductive Sciences, Yale University School of Medicine, New Haven, Connecticut, USA

**Keywords:** premature ovarian insufficiency, POI, ovarian physiology, ovarian sex hormones

## Abstract

Premature ovarian insufficiency is a complex and relatively poorly understood entity with a myriad of etiologies and multisystem sequelae that stem from premature deprivation of ovarian sex hormones. Timely diagnosis with a clear understanding of the various comorbidities that can arise from estrogen deficiency is vital to appropriately counsel and treat these patients. Prompt initiation of hormone therapy is critical to control the unsolicited menopausal symptoms that many women experience and to prevent long-term health complications. Despite ongoing efforts at improving our understanding of the mechanisms involved, any advancement in the field in recent decades has been modest at best and researchers remain thwarted by the complexity and heterogeneity of the underpinnings of this entity. In contrast, the practice of clinical medicine has made meaningful strides in providing assurance to the women with premature ovarian insufficiency that their quality of life as well as long-term health can be optimized through timely intervention. Ongoing research is clearly needed to allow pre-emptive identification of the at-risk population and to identify mechanisms that if addressed in a timely manner, can prolong ovarian function and physiology.

## Introduction

Premature ovarian insufficiency (POI), previously referred to as “ovarian failure”, is characterized by an accelerated truncation of ovarian physiology, chronologically well in advance of the timeline destined for the typical female *Sapiens*. Attrition in the number of residual ovarian follicles and ensuing deficiency in ovarian sex hormones are hallmarks of POI, thus rendering a woman subfertile and estrogen-deficient years, even decades, prior to the normal age of menopause. Subtleties in clinical presentation and a relative lack of population awareness surrounding this condition can lead to a delay in diagnosis and subsequent treatment. Beyond the psychological burden that this diagnosis consistently inflicts, health and well-being of women with POI are sensitive to any delay in the diagnosis and rely on timely initiation of hormone therapy (HT). Without prompt and adequate HT, women may develop severe symptoms and long-term health consequences of estrogen deficiency. One aspect of the confusion among both population and providers stems from the various nomenclatures used to reference this condition. The terms POI, premature menopause, and premature ovarian failure (POF) are synonymous and often are used interchangeably in the literature. Given the continuum of impaired ovarian function and the negative connotation of the word “failure”, the preferred term is POI. In order to remain consistent with recent recommendations for the nomenclature, the term POI will be used in this review.

To better understand the mechanisms underlying early ovarian dysfunction, it is prudent, depending on its mode of onset, to perceive POI as either spontaneous or consequent to a recognized insult such as following surgery (that is, bilateral oophorectomy) or following chemotherapy or radiation exposure. Although the two categories have a similar endpoint of premature and drastic attrition in the ovarian reserve and a paucity of circulating sex hormones, the onset varies between the two as often spontaneous POI is insidious in presentation and delay in diagnosis is common. In contrast, iatrogenic POI is almost always anticipated by both the patient and managing clinicians, and interventions to mitigate symptoms and harness the long-term health risks consequent to the hypoestrogenemia are initiated earlier in the process.

Evidence of hypergonadotrophic hypogonadism in the setting of amenorrhea (primary or secondary) in any woman younger than 40 years clinches the diagnosis of POI; depending on the trajectory of ovarian decline and pace of events, symptoms of hypoestrogenism may or may not dominate the clinical picture. This review will focus on the recent advances in the evaluation and management of POI with the goal of improving preparedness of health-care providers in ensuring optimal care for women afflicted by this complex yet common entity.

## Clinical presentation

The spectrum of symptomatology of POI is highly variable; symptoms can include manifestations of hypoestrogenism as well as additional features that may reflect unique signs of the underlying disorder that is causative of POI ( Table 1). For some women, the first indictor of ovarian insufficiency is reflected by new-onset menstrual irregularities that can range from infrequent to even too frequent menses before amenorrhea eventually sets in. Primary amenorrhea may be the presenting symptom in up to 10% of cases of POI; however, in the vast majority of women diagnosed with POI, menses commence and linger for varying lengths of time following normal pubertal development ^1^. Loss of regular menses for three consecutive months in an otherwise healthy and non-pregnant woman warrants further investigation, and POI should be considered among the differential diagnoses. For other women, distressing menopausal symptoms reflecting an estrogen-deficient state, such as hot flashes, dyspareunia, sleep disturbances, decreased libido, or vaginal dryness, may be the motivation for an initial medical consultation. Notable, however, is that women with primary amenorrhea may never experience menopausal symptoms; specifically, symptoms of hypoestrogenism are rare in those who were never exposed to estrogen. Furthermore, for some women, diagnosis of POI may be discovered only during evaluation for infertility.

**Table 1.** Clinical presentation of premature ovarian insufficiency.

Menstrual abnormalities Missed cycles leading to amenorrhea Sudden onset of secondary amenorrhea Primary amenorrhea
Subfertility/infertility
Menopausal symptoms (hot flashes, vaginal dryness, and sleep disturbances)
Changes in skin pigmentation Vitiligo (autoimmune) Hyperpigmentation-adrenal insufficiency (autoimmune)
Hair loss/alopecia (autoimmune)
Goiter (autoimmune)
Fatigue
Anxiety/Depression

### Induced, often iatrogenic, premature ovarian insufficiency

Bilateral oophorectomy occurs most commonly at the time of hysterectomy for both benign and malignant indications ( Table 2). Since most hysterectomies occur between 35 and 45 years of age, surgical POI is the leading cause of hormone deficiency in pre-menopausal women ^2^. Although the importance of ovarian preservation has received more attention in recent years, more than 200,000 women still undergo bilateral oophorectomy each year in the US ^3, 4^. Similarly, as more women are surviving malignancies treated with gonadotoxic chemotherapy or radiation, the incidence of iatrogenic POI is on the rise ( Table 2) ^5^. The degree of ovarian impairment is dependent on the woman’s age at the time of insult, type of gonadotoxic agent used, and the dose of chemotherapy or radiation used ( Table 3) ^6– 10^.

**Table 2.** Causes of premature ovarian insufficiency.

Spontaneous
*Idiopathic* *Genetic* Turner syndrome (45XO) or mosaic Turner (45X/46XX) Trisomy X (47XXX or mosaic) Fragile X premutation Galactosemia (galactose-1-phosphate uridyltransferase deficiency) Autoimmune polyglandular syndrome (types 1 and 2) Follicle-stimulating hormone receptor mutations 17α-hydroxylase deficiency Aromatase deficiency Blepharophimosis, ptosis, epicanthus inversus syndrome Bloom syndrome Ataxia telangiectasia Fanconi anemia *Autoimmune* *Infections* Mumps oophoritis Tuberculosis, malaria, cytomegalovirus, varicella, and shigella
Induced
Bilateral oophorectomy, bilateral ovarian cystectomies Chemotherapy-primarily, alkylating agents and anthracyclines Radiation-external beam or intracavitary Environmental toxins Pelvic vessel embolization

**Table 3.** Chemotherapy and radiation exposure and risk for premature ovarian insufficiency.

Chemotherapuetic agents and class known to cause gonadotoxicity and premature ovarian insufficiency risk ^7^	Radiation dose and age at exposure as determinants of permanent ovarian damage and premature ovarian insufficiency risk ^8, 9^
**Alkylating agent** Nitrogen mustard Chlorambucil Cyclophosphamide Busulfan Melphalan Dacarbazine	20.3 Gy at birth 18.4 Gy at age 10 years 16.5 Gy at age 20 years 14.3 Gy at age 30 years 6.0 Gy at age 40 or more years
**Anthracycline** Doxorubicin	
**Substituted hydrazine** Procarbazine	

### Spontaneous premature ovarian insufficiency

This condition was first recognized in the 1930s when young women with amenorrhea were discovered to have elevated urinary gonadotropins. It was not until the 1940s that an association was made between the presence of high urinary gonadotropin levels and the classic histologic changes seen in the ovaries of menopausal women ^11^. In the ensuing decades, much has been learnt about the pathophysiology, along with the discovery of interventions that have been transformative in allowing women with POI to lead relatively normal and fulfilling lives.

The trajectory of attrition in the ovarian complement of gametes is at play well before the birth of a female infant, and this natural process of follicular atresia continues throughout the reproductive life span until menopause. However, when this nadir in the ovarian follicle complement is attained prior to the age of 40, it is considered “premature” given that it is 2 standard deviations from the mean estimated age of menopause (50 ± 4 years) seen in the reference population ^12^.

A number of mechanisms can impact on the pace as well as the magnitude of progressive ovarian follicular atresia that characterizes human female gonadal physiology from early *in utero* existence up until menopause. Early ovarian senescence may reflect just an inherently small pool of ovarian reserve or quantity of primordial follicles that a woman is endowed with at the time of gametogenesis. Alternatively, POI may be the result of an accelerated destruction of oocyte complement because of a complex spectrum of disorders with genetic, autoimmune, or toxic underpinnings ( Table 2 and Table 3) ^13, 14^. Spontaneous POI is not an uncommon condition; it is estimated that approximately 0.3% to 1.1% of reproductive-age women experience menopause prematurely ^15^. Among women younger than 40, the incidence of POI steadily increases with advancing age. POI is recognized in 0.01% of women younger than 20, 0.1% younger than 30, and about 1% of woman younger than 40 ^16^.

## Diagnosis

As mentioned earlier, evidence of hypergonadotrophic hypogonadism in the setting of amenorrhea (primary or secondary) in any woman younger than 40 years is consistent with a diagnosis of POI. It is important to appreciate that unlike women with menopause, which marks an irreversible state of ovarian senescence, women with POI retain some degree ovarian function. Thus, fluctuations in ovarian hormones as well as sporadic spontaneous ovulations can be encountered in a small proportion of women with POI, adding complexity to the clinical picture.

A thorough review of medical and family history can provide some insight and guide providers in the process of arriving at a timely and correct diagnosis. Particular attention must be paid to the chronology of events, personal exposures, and family history. A detailed menstrual history that inquires about age of menarche, as well as both frequency and pattern of menses, is useful to determine any menstrual alteration early in the course of events. Medical conditions, medications prescribed, or previous gonadotoxic exposures should be noted since these can directly impact ovarian reserve and function. Of importance, documentation of any endocrinopathies (such as type 1 diabetes mellitus and hypothyroidism associated with Hashimoto’s thyroiditis) should be noted since it is not uncommon for these conditions to be seen in patients with POI. Although tobacco exposure (active as well as passive) has been associated with early menopause and is a recognized detriment to ovarian biology, its relevance for the causation of POI remains unclear ^17, 18^. Information on maternal age at menopause and family history of POI in first- or second-degree relatives may be pertinent, as can a history of mental retardation (particularly so in the male progeny), recurrent pregnancy loss, and history of any known autoimmune or genetic disorders in family members. A thorough review of systems should enquire about symptoms of fatigue and weight fluctuations and focus on, among other things, any involvement of the musculoskeletal and integumentary systems.

Physical examination should focus on stigmata of any underlying disorder (for example, skin depigmentation or hyperpigmentation may reflect underlying autoimmune disorder, as can evidence of a goiter) and on evidence of hypoestrogenism ( Table 1). Although examination is typically unremarkable in the majority of cases of POI, it may reveal evidence of estrogen deficiency marked by lack of or delayed secondary sex characteristics (when POI occurs prior to onset of puberty) or features of genital atrophy. During examination, providers should also look for stigmata that could point to underlying disorder causative of POI, such as cutaneous signs suggestive of an autoimmune conditions or phenotypic hallmarks of a chromosomal disorder (for example, short height, cubitus valgus, and dorsal cervical fat pad are all suggestive of Turner syndrome) ^19^. While signs and symptoms can be suggestive of POI, the diagnosis requires biochemical evidence of hypergonadotropic hypogonadism. Elevated serum follicle-stimulating hormone (FSH) levels (>25 IU/L) on two separate occasions at least one month apart, with concomitant low estradiol (E2) levels (<50 pg/mL), and amenorrhea for at least 4 months in women younger than 40 years of age are collectively required to establish a diagnosis of POI ^18, 20^. Additional laboratory tests can reinforce this impression and at times identify abnormalities that can point to an underlying mechanism (or mechanisms) for POI ( Table 4). Markedly low to undetectable serum levels of anti-mullerian hormone (AMH) in the setting of elevated FSH and suppressed E2 levels reaffirm the impression of POI ^21^. AMH may be particularly beneficial early in the phase of menstrual irregularity since the timing of analysis is independent of timing in a menstrual cycle, whereas FSH levels can fluctuate throughout the cycle and give a false sense of reassurance. A pelvic ultrasound, preferably using a transvaginal approach, can further confirm concerns for ovarian compromise. In appropriate clinical settings, evidence of a thin endometrial echo (<4 mm), small ovarian volumes, and low antral follicle counts (<5) are all consistent with a picture of POI; however, radiological imaging is not essential to establish the diagnosis.

**Table 4.** Diagnostic considerations in evaluation of primary ovarian insufficiency.

Laboratory tests	Rationale
Human Chorionic gonadotropins	Exclude pregnancy
Follicle-stimulating hormone Estradiol	Assess hypothalamic-pituitary-ovarian axis
Anti-mullerian hormone	Assess ovarian reserve
Karyotype, fragile X mental retardation 1 (FMR1) premutation	Evaluate for genetic etiology
Thyroid-stimulating hormone Thyroid peroxidase antibody 21-hydroxylase antibody	Evaluate for thyroid function Quantify risk for thyroid and adrenal dysfunction
Radiologic tests	Rationale
Transvaginal ultrasound	Evaluate antral follicle count to assess ovarian reserve
Dual-energy x-ray absorptiometry scan	Assess bone density

## Etiologies of premature ovarian insufficiency

### Idiopathic

Once POI is suspected, confirmation of this impression requires evidence of hypergonadotropic hypogonadism. A finite list of investigations is advised ( Table 4) with the goal of unmasking a possible etiology (which may have unique relevance for overall health) and quantifying short- and long-term risks (such as fracture risk due to low bone mass). Many underlying disorders may pose unique risks (for example, risk of aortic aneurism in a patient with Turner syndrome and an increased lifetime risk of autoimmune adrenal failure in patients with POI associated with autoimmune thyroid disease). The multiple etiologies of spontaneous POI that are recognized thus far include genetic, autoimmune, infectious, and idiopathic ( Table 2). Most cases of POI, accounting for over 90%, are idiopathic with no discernable cause ^20^.

### Genetic

Genetic influences determine the size of the primordial follicle pool, impact on the rate of follicular atresia, and are a major determinant of eventual age at menopause. With the advent of more sophisticated genetic screening technologies, genetic etiologies for POI can account for about 20% to 25% of cases ^22– 24^. X-chromosome aberrations are the most commonly recognized genetic underpinnings for POI. Although the precise mechanism is not completely understood, it is thought that mutations on the loci of the long arm (q) of the X chromosome that regulate germ cell development and viability may be a contributing factor ^25^. Turner syndrome, which has a phenotype associated with complete or partial monosomy X and affects about 1 in 2,500 females, is the most common genetic condition resulting in POI ^26^. The most frequent chromosome constitution is 45 XO, or complete absence of one X chromosome. Women with 45XO have gonadal dysgenesis, and most cases have only streak ovaries composed of fibrous stromal tissue containing few or no ovum ^19^. About half of patients with Turner syndrome are mosaic, of which the most common chromosome composition is 45X/46XX (15%) while 46XXq or 46XXp deletions account for about 6% ^19, 27^. In patients with partial monosomy X, the exact karyotypic abnormality is relevant to determining the phenotype. Deletions on the short arm (p) of the X chromosome are associated with dysmorphic features such as short stature and congenital malformations, whereas partial or complete deletion of the q arm often manifests in gonadal dysfunction ^27^.

The fragile X mental retardation 1 ( *FMR1*) gene that is located on the q arm of the X chromosome is of particular relevance in the context of POI ^28^. Fragile X syndrome, the most common cause of familial mental retardation, results from an inherited triplet repeat mutation in the *FMR1* gene. The normal number of CGG repeats in the untranslated region of the *FMR1* gene is less than 40. Repeat length between 55 and 200 is termed a premutation, and a length of greater than 200 repeats represents the full mutation ^25^. Interestingly, only those women with a premutation in the *FMR1* gene are at risk for developing POI, whereas those with either normal or full mutation are at no higher risk than the general population. Premutations of the *FMR1* gene are present in as many as 14% to 20% of women with familial POI and are found in up to 2% to 5% of women with isolated POI ^29– 33^. Owing to the increased risk of women with POI harboring the *FMR1* gene premutation, a family history of fragile X syndrome, unexplained mental retardation, tremor/ataxia syndrome, or having an affected child with developmental delays should be solicited. Furthermore, all women with POI who are seeking fertility should be screened for the FMR1 premutation given that spontaneous expansion of the trinucleotide repeat region can be transmitted to offspring in the unlikely event of successful conception, thus increasing the likelihood of fragile X syndrome in the progeny inheriting the full mutation. Male transmission of *FMR1* CGG repeats can also occur, although the transmission tends to be less stable than in women. In males, the CGG repeat can expand, contract, or remain unchanged, and the risk of expansion to full mutation is less frequent than in women ^34^.

Several less common genetic disorders have been associated with POI and should also be considered, particularly when there is a known family history of the disease traits ( Table 2). In the absence of any informative family history, autosomal genetic testing is not currently indicated in the workup of women with POI unless there is evidence or suspicion suggesting a specific mutation ^18^. With all genetic conditions, the patient should be referred to genetic counseling to fully comprehend the transmission pattern and full sequelae of the mutation.

In recent years, attention has focused on genes that are known to play a role in folliculogenesis and ovarian function. Oocyte-specific gene expression is necessary for primordial follicle formation and their subsequent differentiation into primary follicles. Several causative mutations in transcription factors, which regulate oocyte-specific genes, have been implicated in POI ^35^. Examples of POI transcription candidate genes include forkhead box L2 ( *FOXL2*), nuclear receptor subfamily five group A member 1 ( *NR5A1*), newborn ovary homeobox ( *NOBOX*), and factor in germline alpha ( *FIGLA*) ^35– 38^. Similarly, folliculogenesis growth factors, such as bone morphogenetic protein 15 ( *BMP 15*), growth differentiation factor 9 ( *GDF-9*), and inhibin alpha ( *INHA*), which promote follicular maturation and folliculogenesis, are candidate genes. Heterozygous variants of these growth factors have also been associated with POI ^35, 39, 40^.

Apart from the candidate gene approach, cytogenic and genetic linkage studies have been employed to discover a possible etiology of genetic POI. Cytogenic studies, primarily those affecting the X chromosome, have been identified and are closely associated with POI. Monosomy (X0) is the most common, but deletions, duplications, and translocations on the X chromosome have also been detected, primarily within two main loci named *POF1* and *POF2*, respectively ^41, 42^. A second strategy has been to use genetic linkage in combination with positional cloning, a technique most advantageous for monogenic disease ^41^. Using this technique, genes encoding the gonadotropin receptors, follicle-stimulating hormone receptor ( *FSHR*) and luteinizing hormone choriogonadotropin receptor ( *LHCGR*), have been shown to harbor mutations, which results in the POI phenotype. FSH, secreted by the pituitary gland, is vital for the follicular recruitment and growth. Familial studies have shown that mutations in the *FSHR* gene, albeit rare, are associated with amenorrhea and ovarian dysfunction ^43, 44^. The concern with this technique is that the majority of idiopathic POI cases are sporadic; thus, the utility of genetic linkage is nominal.

In efforts to detect novel POI genes, array comparative genomic hybridization (CGH) and genome-wide association studies (GWASs) have been used. With array CGH, reference DNA segments and affected DNA segments are labeled using different fluorochromes. The two samples are subsequently co-hybridized to induce competition and then compared with a given DNA sequence through an array. With this technique, the gain or loss of chromosomes or chromosomal regions, also referred to as copy number variations, between the two groups can be detected ^45^. Any disparity is examined to see whether the affected region has a role in ovarian pathophysiology or reproduction, thus introducing a potential candidate gene. With this modality, several regions of potential candidate genes have been identified; however, no causative mutation has been found ^41^.

More recently, genotyping via GWASs and genome-wide sequencing via next-generation sequencing (NGS) have been applied in efforts to identify genetic variations across the entire human genome that are associated with POI. GWAS and NGS allow assessment of the entire human genome with very detailed resolution and against numerous unrelated individuals ^35^. This evaluation is achieved by using single-nucleotide polymorphisms (SNPs), the most common genetic variations in the human genome, in patients with POI versus unaffected controls ^35^. A single nucleotide difference between two homologous chromosomes is considered polymorphic if both of these differences are found in at least 1% of the chromosomes in interbreeding populations ^35^. SNPs are then used as genetic markers in identifying genetic variants, which can be done either in familial cases or in a large cohort of patients. Although the application of GWASs in POI research has accelerated the identification of novel candidate genes and genetic risk factors, it has not identified any genes that have a significant role in the development of POI. There are only a limited number of GWASs that have been conducted on patients with POI, and the population size has been relatively limited.

Since one of the limitations of GWASs is the use of traditional Sanger sequencing, which can analyze only 700 base pairs per reaction, the use of NGS has proven to be a powerful tool to identify novel genes ^46^. NGS is a high-throughput DNA sequencing technique that can analyze millions of base pairs across the entire exome or genome in a single reaction ^47^. Thus far, the studies of POI using NGS have been limited to primarily inherited POI within families ^46, 48– 50^. These studies detected numerous pathogenic variants in genes associated with the following DNA repair pathways, hormone signaling, genomic instability, immune function, and gonadal development ^50^. For example, with whole-exome sequencing, an analysis of a consanguineous Palestinian family with POI revealed a one–base pair deletion in the stromal antigen 3 ( *STAG3*) gene ^47^. *STAG3* encodes a meiosis-specific subunit of the cohesin ring, which ensures correct sister chromatid cohesion. Murine studies of the *STAG3* knockout supported the POI phenotype ^48^. As evident by the myriad of gene variants identified, without any clear frontrunner, the role that each variant plays in this complex disease is uncertain ^50^.

Lastly, the analysis of microRNAs (miRNAs) has been applied to POI research. miRNAs are small non-coding single-stranded RNA molecules, approximately 18–25 nucleotides in size, which are involved in post-translational gene regulations and RNA silencing ^35, 51^. Although most studies looking at the role of miRNA in POI have been performed in murine models, there have been limited human studies evaluating miRNA within small cohorts of patients with POI ^52^. The first study was performed on three patients with POI and revealed an upregulated miRNA, mir-23a, which is shown to promote granulosa cell apoptosis ^53^. A larger study, in which Chinese women with POI were compared with controls, found 22 upregulated and 29 downregulated miRNAs within the affected subgroup. Upon further evaluation, attention was turned to mir-22-3p because of its regulation of pituitary FSH secretion. These few studies show that miRNA can affect ovarian function and folliculogenesis ^54^. As with all of the previous modalities discussed, larger sample size and evaluation within different ethnicities are indicated to determine the role that miRNA plays in POI.

Although numerous sequencing projects have involved the discovery of causative and candidate genes, the coding mutations found can explain only a minority of cases. Over 50 mutations have been validated in several genes (that is, *FSHR*, *LHCGR*, *NR5A1*, *NOBOX*, *FOXL2*, *FIGLA*, *BMP15*, *NANOS3*, and *STAG3*) via function assays as causative of idiopathic POI, while many other genes can be implicated ^37, 38, 40, 43, 46, 48, 50, 55, 56^. This further supports the notion that POI is a heterogeneous genetic condition that involves the interplay of various genetic alterations and environmental factors, which remain unidentified. Since genes are identified on the basis of statistical significance and not necessarily biological relevance, often little is known about the physiological function of the identified variants. Clearly, more studies using larger cohorts to confirm their relevance and validity are needed.

### Autoimmunity

More than 20% of women with POI will manifest more than one autoimmune condition ^57^. Autoimmune disorders of the thyroid gland (Graves’ or Hashimoto’s disease), seen in 20% to 30% of cases, are the most common autoimmune manifestations associated with POI ^58– 60^. Autoimmune adrenal failure (Addison’s disease) is the second most common autoimmune disorder associated with POI and has the most severe health implications if undetected; it is seen in 3% of women with POI. Less frequently encountered is type I diabetes mellitus at 2.5% ^60^. Screening for anti-adrenal antibodies (specifically, anti-21-hydroxylase-Ab) and thyroid antibodies (thyroid peroxidase and, less commonly, thyroglobulin-Ab) is recommended in women with an unknown etiology for POI or if an autoimmune etiology is suspected ^18^. Respective end-organ function should be performed in women with POI given the high incidence of thyroid and adrenal disorders in this subset of women ^18, 61^. Yearly surveillance of thyroid-stimulating hormone levels should be undertaken in those positive for thyroid antibodies. Similarly, assessment of adrenal function using the adrenocorticotropic hormone (ACTH) stimulation test should be assumed in those testing positive for anti-adrenal antibodies. To perform this provocative test, a baseline level of cortisol is obtained prior to administration of a small dose of ACTH (typically synthetic cosyntropin [1 µg] or an analogous corticotropic agent). The cortisol level produced by the adrenals in response to the ACTH bolus is measured at 30 and 60 minutes and subsequently compared with baseline levels. Cortisol levels should be more than 18 to 20 µg/dL in response to the ACTH stimulation; however, if the cortisol levels remain low, primary adrenal insufficiency is implied. If thyroid and adrenal antibodies are initially negative, there is no indication for repeat testing on a routine basis ^18^. However, should signs or symptoms develop at a future date, a low threshold should be maintained to repeat screening for the suspected endocrinopathy ^18^.

Circulating anti-ovarian antibodies are commonly detected in women with POI with evidence of other autoimmune disorders, and different studies have reported detection rates ranging from 10% to 69% in women with POI ^62, 63^. However, despite this association, testing for anti-ovarian antibodies is of poor prognostic relevance and interpretation is highly variable. Therefore, anti-ovarian antibody testing is not recommended in the evaluation of women with POI.

### Infections

Infectious etiologies have been implicated as a cause of POI, but the true incidence of post-infectious-oophoritis ovarian failure is uncertain. A variety of infections (viral and bacterial), including mumps oophoritis, tuberculosis, malaria, varicella, cytomegalovirus, and shigella, have been associated with POI ^64, 65^. In the majority of cases, once remission of the infectious insult is attained, normal ovarian function ensues ^65^. An infectious etiology should be considered if history relates symptomatology to a potential infectious exposure (that is, travel or sick contact). However, there is no indication to screen for infectious etiologies in a woman with POI without noted risk factors ^18^.

## Sequelae and management of premature ovarian insufficiency

### Menopausal symptoms

Women with POI are susceptible to the gamut of symptoms experienced by the chronologically older cohorts approaching and going through menopause, including the classic menopausal symptoms of hot flashes, night sweats, sleep disturbances, mood instability, and issues of sexuality resulting from vaginal dryness, dyspareunia, and decreased libido. These symptoms can be particularly profound in cases of iatrogenic POI, and severity of bother is often so great that it significantly impacts a woman’s quality of life, psychological well-being, and intimate relationships. Menopausal symptoms in patients with POI are consequent to the premature decline in ovarian follicle number and a dysfunction of the residual follicles with resulting hypoestrogenemia and, to a lesser extent, a decline in ovarian testosterone. Adequate systemic estrogen replacement is critical for symptom control, whereas local estrogen may be required for addressing focal symptoms such as dyspareunia or other genito-urinary symptoms ^1^. Testosterone supplementation as an adjunct to systemic estrogen therapy may be of particular benefit for addressing issues of decreased libido in women following iatrogenic POI, although long-term efficacy and safety of androgens in menopause management remain uncertain ^18^.

### Cardiovascular health

Women with untreated POI have a decreased life expectancy, largely due to cardiovascular morbidity and stroke ^18, 66, 67^. Compared with age-matched controls, women with POI had reduced vascular endothelial function, which is an early precursor to atherosclerosis ^68^. The use of HT for 6 months in this cohort of women significantly improved endothelial function ^69^. Estrogen has beneficial effects on cholesterol metabolism lessening atherosclerotic plaque formation and prevents coronary constriction via catecholamine modulation ^70^. Since estrogen dearth is the culprit, early initiation of HT following onset of menopause has been suggested as a cardio-protective strategy. Although randomized controlled trial data on cardiovascular benefits of HT remain equivocal, there is a consensus of opinion among experts in the field that benefits of initiation and continuation of HT in otherwise healthy women with POI until the average age of menopause (about 51 in the Caucasian population) far outweigh any purported risk ^71^. Despite lacking longitudinal outcome data in young women with POI, the cardio-protective benefits of HT are extrapolated from the data involving post-menopausal women ^18, 72, 73^. Moreover, it cannot be stressed enough how vital it is for women with POI to optimize modifiable risk factors (that is, diet, exercise, and smoking) to ensure cardiovascular health.

### Bone health

Peak bone mass is attained by age 30 in women, and estrogen is fundamental to the bone accrual process ^68^. Deprivation of estrogen consequent to POI is a known fracture risk in later life ^18, 74– 76^ and this risk can be mitigated with use of HT ^68, 74^. When compared with age-comparable menstruating women (mean age of 32 years), not only did those with POI have significantly lower bone mineral density (BMD), but 21% of those with POI had BMD z-score of less than −2.0, putting them at significant lifetime fracture risk ^68, 75^. Assessment of BMD may be useful and should be considered for women with POI; after the initial assessment, repeat BMD assessments can be deferred for those choosing to continue HT until the population’s average age of menopause (51 years) or until the recommended age of 65 for all women ^18^. Evidence of low bone mass should be deemed an indication for prioritizing initiation of HT in any woman diagnosed with POI, and in these patients, serial monitoring of bone mass (within 2 to 5 years of initiating HT) is warranted. HT may have an even greater impact on women younger than 30 years of age, particularly if they have not yet attained peak bone mass ^77^. Lifestyle modifications such as weight-bearing exercises, optimization of dietary calcium intake and vitamin D status, and avoidance of cigarette use can all contribute to help preserve bone health ^18^. Declining BMD despite adequate HT dosing requires consideration of secondary causes of bone loss and may dictate consideration for use of non-hormonal anti-resorptive agents for fracture risk reduction ^18^. In the setting of low but stable BMD on HT regimen, pharmacologic agents such as bisphosphonates are generally avoided given the long half-life of these medications. The potential risk for fetal toxicity in future conceptions (either spontaneous or through use of donor eggs) is a concern in this young population ^71^. 

### Cognition

Premature deprivation of estrogen, such as following bilateral oophorectomy undertaken in pre-menopausal women, has been associated with neurocognitive decline. Women who underwent surgical menopause consequent to bilateral oophorectomy before natural menopause had an increased risk of cognitive decline and Alzheimer’s disease neuropathy, in particular neuritic plaque formation ^2, 78^. Of note, the earlier the age of surgical POI, the more rapid the cognitive decline ^79^. In contrast, women who were started on HT within 5 years of surgical menopause and continued treatment for 10 years showed decreased cognitive decline, suggesting protective effects of HT on neurocognition; notably, however, Alzheimer’s disease neuropathology was not reversed ^2, 78^. Appropriately designed studies are needed to understand whether women with POI are similarly at risk for neurocognitive decline as those rendered hypoestrogenemic following bilateral oophorectomy. Until then, the surgical POI data offer a logical benchmark to guide use of HT by women with POI until the age of natural menopause as a strategy to minimize risk of chronic disorders, including osteoporosis and premature cardiovascular disease, as well as for neurocognitive well-being ^18, 80^.

### Emotional health

The diagnosis of POI is most often unexpected, creating psychological havoc for the afflicted young women. A realization of compromised fertility, fear of premature aging, and a perception of being different than their peers can all be emotionally overwhelming. Women with POI report reduced self-esteem, increased social anxiety and shyness, and symptoms of depression when compared with normal peers ^81^. Early psychological counseling should be advocated, and made easily accessible, for any woman stepping into menopause prematurely. Frequent visits with the woman’s multi-disciplinary team should be arranged to provide continued observation of her coping strategies and to address unmet needs.

### Infertility

The rapid and unanticipated truncation in a woman’s reproductive life span is among the most distressful sequela of POI diagnosis, particularly for those women who have yet to embark on planning a family. Whereas fertility is markedly compromised, infertility in the setting of POI may not be absolute. Up to 25% of women with POI may spontaneously ovulate, and 5% to 10% will conceive and deliver after being diagnosed with POI ^1, 82^. Despite marked advances in the field of reproductive medicine in recent years, there are no interventions that can reliably improve residual ovarian reserve parameters or any treatment other than use of donor eggs which can improve conception rates in women with POI ^18, 83^. Women with POI should be reassured that spontaneous pregnancies from idiopathic POI do not show any higher obstetric morbidity or neonatal risk as compared with the general population ^18^. Conversely, those not interested in future fertility should be made aware that, despite the recognized reproductive compromise, spontaneous resumption of ovarian activity and thus unwanted conceptions can occur. Therefore, contraceptive strategies should be recommended in those women who wish to avoid pregnancy.

Women with POI who are identified with chromosomal aberrations (such as balanced translocations or Turner mosaicism) or single-gene disorders such as *FMR1* premutation carrier state should undergo preconception counseling to better understand the risks to their progeny and of miscarriage in the event that successful conception is achieved; based on risk assessments, many may choose to consider use of donor eggs to maximize their chances for a healthy pregnancy. Those who are diagnosed with Turner syndrome (XO or mosaic XX/XO karyotype) hold unique pregnancy risks due to the high rate of cardiac abnormalities seen in this population and pregnancy-related escalation in risk for catastrophic cardiovascular events such as aortic dissection. Evaluations by a perinatologist and cardiologist are essential given that any evidence of aortic root abnormalities, common in this population, is a contraindication to pregnancy. Depending on the length of the CGG repeats, male children of women who harbor the FMR1 premutation may be at an increased risk of being affected by fragile X mental retardation. Use of *in vitro* fertilization (IVF) with preconception genetic diagnosing can minimize transgenerational transmission of identifiable genetic mutations in families that are deemed at an increased risk of passing genetic mutations to future progeny.

For women with diagnosed or suspected POI for whom fertility is a priority, prompt referral to a reproductive endocrinologist is recommended. For those with established POI, while the opportunity for fertility preservation is missed, assisted conception by IVF using donor gametes or embryos provides the patient with the best option for biological parenting. Oocyte donation cycles generally result in high pregnancy rates given that the success rates are attributed to the age of the preselected and young oocyte donor. Alternatively, some couples prefer to pursue adoption to complete their families. All options should be offered to couples with a clear appreciation that this topic is sensitive and decisions should be individualized.

In recent years, efforts have focused on pre-empting those who may be at risk for POI. In the select few women who can either anticipate the ovarian dysfunction (that is, prior to gonadotoxic treatments or surgery) or discern the diminished ovarian reserve prior to profound cessation of ovarian function, salvage of potential for future fertility may be possible. Cryopreservation of oocytes, embryos, or ovarian tissue is a strategy that can preserve future fertility in select women. Although embryo cryopreservation represents the gold-standard strategy for female fertility, this option is limited to women who are partnered or are willing to commit to donor sperm source. For the single woman, oocyte cryopreservation is a reasonable option, although the likelihood of a reasonable egg yield in the POI population or for those deemed at risk for POI is slim. Anecdotal success has been achieved with cryopreservation of ovarian cortical tissue followed by orthotopic autotransplant (graft into the pelvis); however, this procedure is still considered experimental. Notably, applicability of this technique in the POI population has not been studied and should be offered only at institutions with institutional review board oversight ^84^.

For women anticipating receiving pelvic radiation for management of an underlying disorder, such as cervical cancer, where radiation-induced POI is almost inevitable, surgical transposition of the ovaries out of the pelvis can limit radiation exposure and may serve to preserve gonadal function. For women anticipating undergoing chemotherapy with gonadotoxic agents, other than the fertility preservation options of egg, embryo, or ovarian tissue cryopreservation, suppression of ovarian function pre-chemotherapy with use of gonadotropin-releasing hormone analogs is commonly employed. Efficacy data on the magnitude of fertility preservation achieved using this strategy, however, are conflicting. Until efficacy is clearly demonstrated, proven, and established, fertility preservation therapies should remain first-line options ^85^.

An exciting area of research to improve gamete yield from ovarian tissue in women with POI has recently been reported that uses *in vitro* activation (IVA) followed by auto-transplantation of ovarian tissue. In women with POI one ovary was removed and IVA achieved through activation of the AKT pathway in the few dormant follicles that were found within the ovarian tissue. The ovary was re-transplanted and residual follicles were seen to be active in 6 of the 14 patients (43%), and following IVF with oocyte retrieval, a pregnancy was achieved ^86– 88^. More studies are needed to determine the efficacy of the IVA treatment protocol and whether this will be a useful tool in the future for women with POI.

An area of research that has the potential for revolutionizing the course of POI is that of stem cell technology. Therapeutic potential of hematopoietic and mesenchymal stem cells in humans has progressed over recent years, and much effort has focused on establishing the existence of oogonial stem cells (OSCs), challenging the central dogma that women are born with a fixed number of oocytes that steadily declines until menopause is reached. Indeed, a number of investigators have now reported success in identifying OSCs in experimental models allowing postulation that mitotically active OSCs may also be present in human ovarian cortex ^89– 93^. OSCs have been detected in women and have been able to form oocyte-like structures in a xenotransplant model ^92, 94^; however, the physiological relevance of OSCs in human female remains unclear ^91^. OSC research is a primitive yet exciting area of research that has the potential to revolutionize reproductive medicine, particularly in women with POI or age-related ovarian decline. 

## Hormone treatment in the management of premature ovarian insufficiency

In the past two decades, the role of HT in menopause management has undergone stringent inquisitions, and practice of menopause management has undergone transformative changes driven by myriad of interpretations and misinterpretations of data from the Women’s Health Initiative (WHI) hormone trials ^95^. Notably, the two seminal WHI hormone trials (estrogen alone for women who had previously undergone hysterectomy and estrogen plus progestin trial for those with intact uteri) were undertaken in an elderly and predominantly asymptomatic population of women who were at advanced stage of menopause. The studies were terminated prematurely because of concerns that potential benefit was exceeded by net harm, given the studies did not establish that HT offered cardiovascular benefit (primary endpoint). The fate of the WHI hormone trials was erroneously perceived by practitioners and patients alike as a judgment against safety and place of HT for all. That HT-related concerns observed in an aged and relatively asymptomatic population of menopausal women, who were well established in menopause (WHI population), were extrapolated and deemed applicable to the young and healthy, perimenopausal and early menopausal women (the vast majority of women, who are in need of HT to improve their quality of life) can only be deemed as a tragic misinterpretation of the then-existing data, which has caused a disservice to multitudes of symptomatic women. In recent years, driven as much by introspection and re-analyses of existing data as by reassurance offered by newer clinical trials, the pendulum of opinion and practice has shifted toward moderation and a commonsense approach to use of HT. Most importantly, a vociferous consensus exists in support of using HT in women with POI, both for symptom management and for mitigating burden from chronic disorders that are directly impacted by premature loss of estrogen (such as osteoporosis, cardiovascular, and neurocognitive disorders) and that have been previously highlighted in this review. The goal of HT in POI population is to mimic endogenous levels of sex hormones, which equates to a serum E2 level of about 100 pg/mL, and once initiated, HT should be continued until the age of natural menopause (average age of 51 years) ^68^. Despite the consensus and clarity in safety and recommendation for offering HT to women with POI, an assessment of HT use among this population revealed that more than 52% of women with POI never took HT, started HT years after the diagnosis was established, or discontinued HT before the age of 45 or that a combination of these was the case ^68, 96^. Untreated or inadequately treated women with POI must be perceived at an increased risk of long-term morbidity, which could have been averted if addressed in a timely manner proximate to time of diagnosis.

### Post-pubertal premature ovarian insufficiency

Given the young age at time of ovarian insufficiency and symptom severity experienced by women with POI, this population often requires higher doses of systemic estrogen than is commonly used for managing symptoms in older menopausal women. In deciding on the choice of HT, consideration should be given to dose, route, and regimen; while estrogen alone is optimal for managing symptoms in women who have undergone hysterectomy, the addition of progestin must be incorporated to estrogen therapy in those with an intact uterus. Not only is the thromboembolic risk related to estrogen use suggested to be lessened with transdermal compared with oral route of administration, transdermal dosing is recognized to provide better sustained circulating levels of estradiol compared with oral intake and this itself may offer some advantage for symptom control. The typical starting formulation for women with POI includes transdermal estradiol in a daily dose of 100 μg, oral estradiol in a dose of 1 to 2 mg/day, or conjugated estrogen at a dose of 0.625 to 1.25 mg daily. Patients must be advised to maintain a symptom chart, and hormone dose should be adjusted to achieve a level of symptom control that translates to an optimized quality of life.

Oral combined hormonal contraceptives are an alternative for symptom management and may be psychologically preferred by some young and symptomatic women with POI. It should be noted that combined hormone contraceptives provide supratherapeutic levels of estrogen and progesterone given that the intent of the medication in normal, cycling woman is to suppress ovulation. Therefore, for women who are seeking potential for fertility and willing to avail the slim possibility of spontaneous ovulation, use of hormonal contraceptive regimen should be avoided. If this method is selected, it may be beneficial to avoid the hormone-free placebo week in efforts to minimize the likelihood of recrudescence of symptoms of hypoestrogenemia during the hormone-free window. It should be noted that, in women with POI, oral contraceptive pills are not as efficacious in preventing pregnancy and this is likely due to the persistent elevated serum gonadotropin levels detected within this subgroup. If contraception is desired, barrier contraceptive measures and intrauterine devices are recommended.

In all women with a uterus, progesterone should be added to the estrogen regimen to minimize the untoward risk of endometrial hyperplasia or endometrial cancer, which is a recognized sequel to prolonged use of unopposed estrogen ^97^. Both natural progesterone and synthetic progestins are available in a wide array of formulations (oral tablets, vaginal creams/tablets, intramuscular injection, and intrauterine devices) and regimens (continuous and cyclic). Common formulations that are used for endometrial protection in HT users include medroxyprogesterone acetate 10 mg daily for 12 days each month and oral or vaginal micronized progesterone 100 mg daily or 200 mg daily for 10 to 12 days per month. To date, the only study evaluating HT in women with POI is the National Institutes of Health Intramural Research Program, which used transdermal E2 (100 μg/day) with oral medroxyprogesterone acetate (10 mg/12 days), which was well tolerated ^74^. While there are some advantages to preferential use of micronized natural progesterone (reduced breast cancer risk in some studies and some benefit for improved sleep), the strongest evidence of endometrial protection is for oral cyclic medroxyprogesterone acetate ^18^. Although the efficacy of oral micronized progestin or progestin-containing intrauterine contraceptive devices to effectively induce endometrial decidualization has not been systematically evaluated as a component of HT for women with POI, these formulations are increasingly being incorporated in menopause management, and future trials should specifically explore their value for women with POI.

### Prepubertal premature ovarian insufficiency

In some young women, onset of POI predates onset of puberty; overt stigmata of failure of ovarian estrogenization include lacking secondary sex characteristics (that is, thelarche), retarded skeletal growth, and primary amenorrhea. Although Turner’s syndrome (XO) is the prototype for prepubertal POI, any condition that results in complete ovarian failure during childhood (such as whole body radiation for bone marrow transplant) would have similar consequences. In keeping with pubertal physiology wherein a gradual escalation in circulating estrogens predates menarche by a few years, for a patient in whom POI strikes prior to puberty, the goal of HT is to achieve exposure to increasing estrogen levels in a progressive fashion over months, starting with very low doses of 17-β estradiol. Typical regimens include 6.25 µg/day of 17-β estradiol transdermally or micronized oral estradiol 0.25 mg/day ^18^. The 17-β estradiol is gradually increased every 3 to 6 months over the course of 2 years until an adult dose is reached or spontaneous menses ensues, at which point cyclic progesterone regimen is added to ensure endometrial protection similar to the one discussed under management of post-pubertal patients. Oral micronized progesterone 100 to 200 mg/day for 12 to 14 days is the most common regimen used ^98^. It should be noted that use of a hormone contraceptive formulation as HT strategy is to be avoided in these young girls until estrogen alone–induced puberty has been completed as premature use of combined hormone regimens can result in tubular breast formation ^26^.

## Summary of current perspective and future goals

Despite the advances in our understanding of POI, the vast majority of cases remain idiopathic, and there is no clear etiology to explain the phenotype. Regardless of the etiology, POI is a state of estrogen deprivation that holds both short-term and life-long implications for health and for psychosocial well-being. The magnitude of ongoing research efforts as well as direction of some novel quests discussed in this review should offer hope for those afflicted as well as invigorate those who are engaged in caring for this unique population. While awaiting the blossoming of a promise that tissue-specific stem cell salvage and therapy may offer, timely initiation of HT and a multi-disciplinary approach must be recognized as cornerstones to effectively manage this complex entity to ensure that physical, psychological, and emotional challenges that result from POI diagnosis are met and short-term and long-term well-being of these young women is optimized.
